# Lightweight convolutional neural network for aircraft small target real-time detection in Airport videos in complex scenes

**DOI:** 10.1038/s41598-022-18263-z

**Published:** 2022-08-25

**Authors:** Weidong Li, Jia Liu, Hang Mei

**Affiliations:** College of Information Science and Engineering, Henan University of Technology, Zhengzhou, 450001 China

**Keywords:** Electrical and electronic engineering, Information technology, Computer science

## Abstract

Airport aircraft identification has essential application value in conflict early warning, anti-runway foreign body intrusion, remote command, etc. The scene video images have problems such as small aircraft targets and mutual occlusion due to the extended shooting distance. However, the detection model is generally complex in structure, and it is challenging to meet real-time detection in air traffic control. This paper proposes a real-time detection network of scene video aircraft-RPD (Realtime Planes Detection) to solve this problem. We construct the lightweight convolution backbone network RPDNet4 for feature extraction. We design a new core component CBL module(Conv (Convolution), BN (Batch Normalization), RELU (Rectified Linear Units)) to expand the range of receptive fields in the neural network. We design a lightweight channel adjustment module block by adding separable depth convolution to reduce the model’s structural parameters. The loss function of GIou loss improves the convergence speed of network training. the paper designs the four-scale prediction module and the adjacent scale feature fusion technology to fuse the adjacent features of different abstract levels. Furthermore, a feature pyramid structure with low-level to high-level is constructed to improve the accuracy of airport aircraft’s small target detection. The experimental results show that compared with YOLOv3, Faster-RCNN, and SSD models, the detection accuracy of the RPD model improved by 5.4%, 7.1%, and 23.6%; in terms of model parameters, the RPD model was reduced by 40.5%, 33.7%, and 80.2%; In terms of detection speed, YOLOv3 is 8.4 fps while RPD model reaches 13.6 fps which is 61.9% faster than YOLOv3.

## Introduction

Aircraft detection on the airport surface is vital for intelligent airports and remote towers. It can automatically identify aircraft in airport images and assist tower controllers in ensuring flight safety. It is essential in daily aircraft conflict early warning, runway intrusion prevention, and remote command-application value^1^. Cameras usually capture surface aircraft images in terminals and towers. Compared with remote sensing aircraft images with complete contours and a single attitude, the surface aircraft images have problems such as mutual occlusion of Aircraft and small Aircraft due to long shooting distances, limiting the traditional methods based on sliding windows^2^. Object detection based on deep learning is one of the most critical tasks in computer vision, such as semantic segmentation^3–5^, image extraction^6,7^, target tracking^8–10^, etc.Target location recognition in complex environment is widely used in face recognition^11,12^, traffic scene detection^13,14^, intelligent video surveillance^15,16^, remote sensing measurement^17,18^, space early warning^19,20^ etc. Unlike the traditional target detection algorithm, the target detection algorithm based on a convolutional neural network uses existing data to automatically learn target features, which has a better expression effect than HOG^21^, SIFT^22^, LBP^23^, and other features can adapt to diverse backgrounds and target types. Still, owing to the dynamic changes in the airport scene, such as temperature, humidity, air pressure, visibility and other complex environments, detecting aircraft on the airport scene is challenging. Currently, the most advanced target detection algorithm is applied to the aircraft detection task on the airport surface, dramatically improving the detection accuracy and speed of the aircraft on the airport surface. However, the detection network model is generally complex because of the problems of small aircraft targets and mutual occlusion between aircraft in the video images of the airport scene. That is not easy to meet the real-time detection requirements in airport air traffic.

In response the above problems, this paper proposes a lightweight neural network RPD for detecting small aircraft objects in complex airport scenes. Our main contributions are summarized as follows.

- We construct a novel deep convolutional feature extraction network, which can help us obtain richer semantic information about the original image, ensure the detection accuracy of the network model, and minimize the computational parameters of the feature extraction network.
- We design an adjacent-scale feature fusion module it can effectively identify aircraft and their location information using four-scale feature prediction.
- We have optimized the loss function. It can correctly guide the convergence direction of the RPD model and can effectively improve the convergence speed of RPD training.

## Related works

The current mainstream target detection methods mainly include the Faster-RCNN^24^ series based on region proposal and the series based on regression algorithm YOLO^25–27^, SSD^28–32^, etc. Compared with traditional methods, the detection accuracy and speed are improved. Still, there are shortcomings, such as many regional proposal boxes, which lead to a large amount of model calculation and ample storage space. The training process of the target detection algorithm requires high-performance GPU support, which is challenging to meet the real-time requirements^33^, especially on embedded devices with weak computing power. Especially on embedded devices with weak computing power. It is not easy to achieve real-time applications^34^. Zhang^35^ et al. proposed a lightweight deep learning model Slimyolov3, which solved the problem that the deep learning model has many parameters and cannot be deployed on the embedded side. Still, its accuracy is poor in small target detection scenarios and cannot be widely used. The aircraft target detection method based on a neural network has achieved high accuracy. Still, its operation on embedded devices with small video memory and memory is restricted with the continuous improvement of the performance of the neural network model and the increase of model parameters and calculation.

When deploying a target detection model in an aviation scene, we need to consider the textcolorredmodel’s computational complexity, parameter quantity and the detection accuracy. MobileNet is a lightweight convolutional neural network proposed by Google in 2017 and subsequently developed into three versions, MobileNetv1^36^, MobileNetv2^37^, and MobileNetv3^38^. Compared with the traditional convolutional neural network, it reduces the model parameters and the amount of computation while ensuring detection accuracy as much as possible. GhostNet^39^ was proposed by Huawei and ShuffleNet^40,41^ and SqueezeNet^42^ presented by Questyle Technology, etc. Building a new network model improves detection accuracy while reducing the model parameters, which is very useful for mobile deployment. Great significance. In addition, deep learning model compression and acceleration technology is also an essential direction in developing network models. Commonly used methods include model pruning^43^, network parameter quantization^44^ and parameter optimization of existing network models. Model pruning adopts structured pruning technology to remove the weights of redundant channels in the model. After pruning, the model can bring acceleration effects on general hardware and improve the efficiency of network operation^45^. Network parameter quantization minimizes the space required for network weight storage by reducing the model detection accuracy. YOLOX^46^ is optimized based on YOLO by combining model pruning and network parameter quantization technology. Although the parameter calculation amount is reduced, they cannot effectively identify small target detection in complex scenes.

In contrast to the previous models, We propose a lightweight neural network RPD for detecting small objects in airport video in complex scenes. Constructing an RPDNet4 deep convolutional feature extraction network, designing an adjacent scale feature fusion module, and using four-scale feature prediction can effectively identify aircraft and their positions information. The effectiveness of the proposed model is verified by comparing it with YOLOv3, Faster-RCNN, SSD, YOLOX-Tiny, and YOLOX-Nano through the Zhengzhou Xinzheng Airport aircraft image dataset experiment.

## Methodology

### Build the RPD network

**Figure 1:**
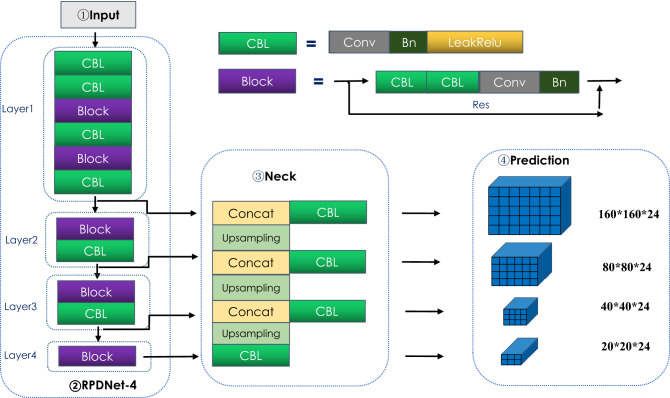
RPD detection framework.

The target detection network proposed in this paper is shown in Fig. 1. It includes four parts: (1) the mage input module, which performs preprocessing operations such as zooming, panning, and random cropping on the input image; (2) the Feature extraction network module (RPDNet4). Among them, CBL and Block are the basic modules of this network. CBL consists of Conv (convolutional layer), BN (batch normalization), and Relu activation function. Block consists of two CBL modules, convolutional, batch normalization composition. (3)the Neck module, which improves the expressive ability of features, in which Concat indicates that image features of different levels complete feature information fusion through downsampling; (4)the Prediction module, used to predict the target: perform 160*160, 80*80, 40*40, 20*20 4-scale target prediction classification and positioning.

#### Feature extraction backbone network

The input image is subjected to preprocessing operations such as zooming, panning, and random cropping to improve the detection accuracy of small objects and mutual occlusion in the airport video scene. Such as formula (1) and formula (2):

1 $$\begin{aligned} f_{n}= F_{n}\left( f_{n-1}\right) , n>0 \end{aligned}$$

2 $$\begin{aligned} Y= G\left( g_{i}\left( f_{i}\right) , \ldots , g_{n}\left( f_{n}\right) \right) , n>i>0 \end{aligned}$$

In formula (1), $f_n$ represents the feature map of the nth layer, and Fn represents the nonlinear mapping relationship between the feature map of the previous layer and the current one. The primary operations are convolution, batch normalization, nonlinear activation function, Etc. When n=1, $f_0$ represents the input image; $g_i$ represents the channel feature pruning and aggregation spatial dimension feature decomposition operation on the *i*-th feature image, *G* represents the feature fusion process, and *Y* represents the feature fusion result.

**Figure 2:**
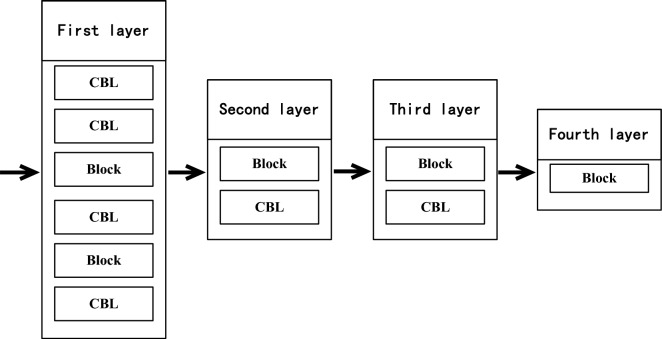
Feature extraction layers.

The feature extraction network (RPDNet4) sets four feature extraction layers according to the direction from input to output. Figure 2 shows the first feature extraction layer, the second feature extraction layer, the third feature extraction layer, and the fourth feature extraction layer. Floor:

The first feature extraction layer includes a first convolution module, a second convolution module, a first residual module, a third convolution module, a second residual module, and a fourth convolution module, which are arranged in sequence from input to output.

The second feature extraction layer includes a third residual module and a fifth convolution module arranged in sequence from the input to the output direction.

The third feature extraction layer includes a fourth residual module and a sixth convolution module arranged in sequence from the input to the output direction.

The fourth feature extraction layer includes a fifth residual module.

**Figure 3:**
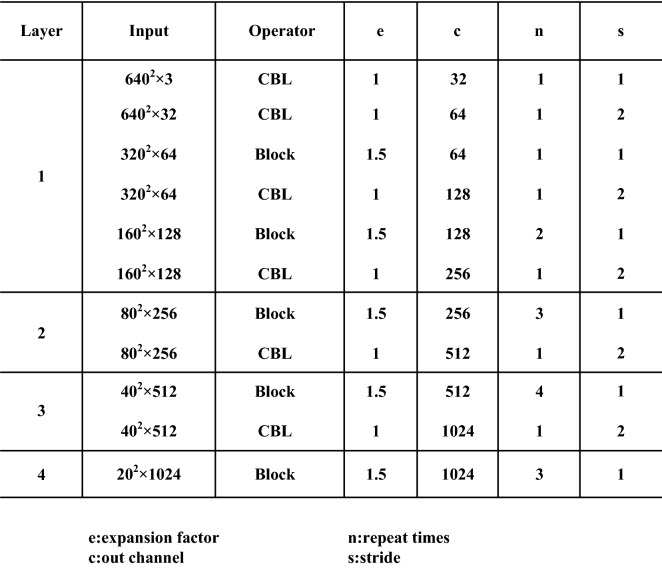
Feature extraction network.

The specific parameter settings of the feature extraction layer are shown in Fig. 3. For the feature extraction layer layer1, the parameter setting steps are as follows.

1. Pass a frame of 640*640 3-channel input image through the CBL module once (n is 1), the step size s is 1, the number of output channels c is 32, the channel expansion factor e is one by default, and the output is 640*640*32 tensor.
2. Using the output result of step 1, after a CBL module (n is 1), the step size s is 2, the number of output channels c is 64, the channel expansion factor e is one by default, and the output is a 320*320*64 tensor.
3. Using the output result of step 2, after a CBL module (n is 1), the step size s is 2, the number of output channels c is 64, the channel expansion factor e is 1.5, and the output is a 320*320*64 tensor.
4. Using the output result of step 3, after a CBL module (n is 1), the step size s is 2, the number of output channels c is 128, the channel expansion factor e is one by default, and the output is a 160*160*128 tensor.
5. Using the output result of step 4, after two block modules (n is 2), the step size s is 1, the number of output channels c is 128, the channel expansion factor e is 1.5, and the output is 160*160*128 tensor.
6. Using the output result of step 5, after one block module (n is 1), the step size s is 2, the number of output channels c is 256, the channel expansion factor e is one by default 80*80*256 tensor.

The feature extraction layers Layer2, Layer3, and Layer4 are consistent with the parameter setting steps of the feature extraction layer Layer1

(1)CBL module

To obtain the richer semantic information of the original images, we need to increase the convolution kernel’s size to expand the range of the receptive field in the neural network. However, a larger convolution kernel size will increase the model calculation parameters. VGGNet^47^ found that the receptive field range of the extensive convolution kernel mapping can be achieved by stacking multiple convolution kernels and using a 3 $\times$ 3 size convolution kernel instead of a 5 $\times$ 5 size volume. The accumulation kernel dramatically reduces the number of model parameters. Based on the idea of VGGNet, this paper designs a new CBL module, consisting of Conv (convolutional layer), BN (batch normalization), and Relu activation function. The convolution kernel is set to 3 $\times$ 3 and 1 $\times$ 1, and the input image is first after a 3 $\times$ 3 convolution operation, the data batch normalization (BN) processing is performed, and the formula is as follows:

3 $$\begin{aligned} \mu _{\beta }= \frac{1}{n} \sum _{i=1}^{n} x_{i} \end{aligned}$$

4 $$\begin{aligned} \sigma _{\beta }^{2}= \frac{1}{n} \sum _{i=1}^{n}\left( x_{i}-\mu _{\beta }\right) ^{2} \end{aligned}$$

5 $$\begin{aligned} x_{i}^{\prime }= \frac{\left( x_{i}-\mu _{\beta }\right) }{\sqrt{\sigma _{\beta }^{2}+\varepsilon }} \end{aligned}$$

6 $$\begin{aligned} y_{i}= \gamma _{i} * x_{i}^{\prime }+\beta _{i} \end{aligned}$$

$\mu _{\beta }$ represents the sample mean, where $x_i$ is the *i*-th sample, $\sigma _{\beta }^{2}$ represents the sample variance, $x_{i}^{\prime }$ represents the sample normalization processing result, $y_{i}$ different scales and bias Shift operation $\beta _{i}$ on $x_{i}^{\prime }$.

To enhance the nonlinear expression ability of the neural network and prevent the gradient explosion of the backpropagation of the network, and speed up the convergence speed of the network, the nonlinear function Relu is introduced as the excitation function, and the formula is as follows:

7 $$\begin{aligned} \text {Relu}=\min (6, \max (0, x)) \end{aligned}$$

(2)Block module

**Figure 4:**
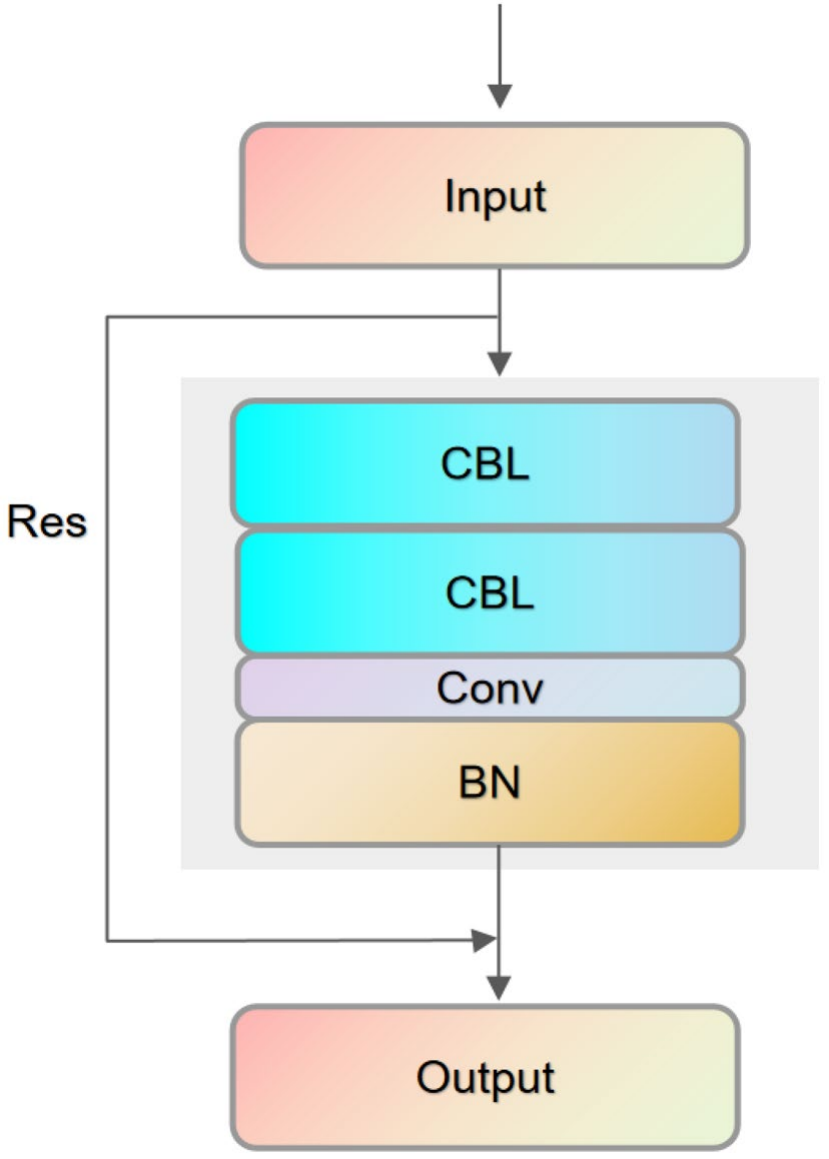
Block.

To ensure the accuracy of network detection and minimize the computational parameters of the feature extraction network, we designed a lightweight channel adjustment module Block, whose structure is shown in Fig. 4. When the input sample passes through the first CBL module, the convolution kernel size is set to 1 $\times$ 1, aiming to map the image features from low-dimensional to high-dimensional space. Factor E is used to expand the dimensional space in Fig. 3. When the features are input to the second CBL module, the high-dimensional spatial convolution is decomposed into a depthwise convolution in a low-dimensional space and a point-by-point convolution that modifies the number of channels using a 3 $\times$ 3 depthwise separable convolution^48^. The convolution of high-dimensional space is decomposed into the depth convolution of low-dimensional space and the pointwise convolution of modifying the number of channels. A convolution kernel is assigned to each channel in the convolution process. Reducing the amount of network calculation and ensuring the network’s complexity and effectiveness, the shrinkage factor e is set to 1.5, and the step size s is set to 2. To maintain the balance of the image feature map size; finally, a residual Res^49^ is added between the input and output connection. The residual Res formula is as follows:

8 $$\begin{aligned} \mathrm {F}(\mathrm {x})=\mathrm {H}(\mathrm {x})-\mathrm {x} \end{aligned}$$

Where *x* represents the input feature, H(x) represents the neural network learning feature, and F(x) represents the output result after the residual connection. When the residual F(x) = 0, the block module only does the identity mapping, which can keep the network performance unchanged. However, in the experiment, the residual F(x) is not 0, so the block module can continuously learn new features and better performance. Using residual connections can significantly preserve the spatial gradient structure, solve the phenomenon of gradient fragmentation, and facilitate network backpropagation.

### Adjacent scale feature fusion and prediction

**Figure 5:**
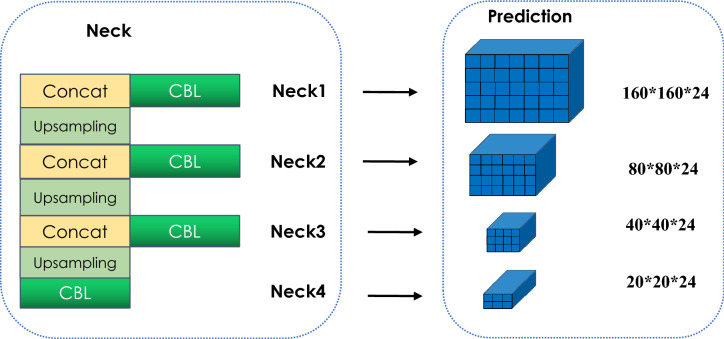
Neck and prediction.

The adjacent scale feature fusion (Neck) module (Fig. 5) is based on the feature extraction network RPDNet4. It adopts a serialized bottom-up structure design to fuse features of different abstraction levels to adjacent features. The Neck module performs upsampling three times in total. Layer4 is used as the starting feature map. After the CBL module, the feature map Neck4 is obtained, and then it is subjected to 2-fold upsampling, batch normalization, and merged with Layer3 for feature fusion. After the CBL module, the feature map Neck3 is obtained. The method obtains the feature maps Neck1 and Neck2 and appends a CBL block to each merged map to generate the final feature map. The final output feature map has four scales of 160*160, 80*80, 40*40, and 20*20, corresponding to Layer1, Layer2, Layer3, and Layer4 with the same spatial size, respectively.

The Prediction module uses the 1 $\times$ 1 convolution operation for Neck1, Neck2, Neck3, and Neck4 instead of a real connection, to complete 4-scale target classification and positioning.

#### LOSS function

Using the Iou Loss loss function to test the lightweight target detection network RPD, the predicted aircraft target position significantly deviates from the accurate position. The common Iou Loss only focuses on the intersection ratio between the predicted and actual frame. When intersecting, the value of Iou Loss is 1, and the network is difficult to converge. When two boxes intersect, the value of Iou Loss is also directional, which cannot guide the network to converge correctly. Therefore, Shortening the centre distance between the target and the natural frame can better reflect the actual deviation between the target and the natural frame. This paper uses GIou Loss^50^ to add a penalty based on the original Iou Loss Item to solve the problem, such as formula (9). It can guide the convergence direction of the network when Iou Loss does not play a role in monitoring and can effectively improve the convergence speed during network training.

9 $$\begin{aligned} {\text {loss}}_{\text{ loc } }=1-\frac{\left| s_{p r e} \cap s_{\text{ true } }\right| }{\left| s_{p r e} U s_{\text{ true } }\right| }+\frac{A_{c}-A_{u}}{A_{C}} \end{aligned}$$

Among them, $s_{p r e}$ represents the predicted target bounding box area, $s_{true }$ represents the natural target bounding box area, ${A_{C}}$ represents the minimum area enclosed by $s_{p r e}$ and$s_{true}$, and $A_{u}$ represents the area of the intersection of $s_{p r e}$ and$s_{true }$. $loss_loc$ represents the position deviation between the predicted target bounding box and the actual target bounding box.

## Experiment

### Experimental environment and dataset

The experimental environment is Ubuntu 20.04 operating system, Intel$\circledR$ Core$^{\hbox {TM}}$ i9-9900K processor, 32 GB memory, GPU is RTX 2080Ti*2, and the deep learning framework uses Pytorch1.8 and general parallel computing architecture CUDA11.1. Regarding training strategy, the Batch Size is 16, the training data set is iteratively trained 300 times in total, the initial learning rate is 0.001, and the learning decay rate is 0.92. In order to further optimize the model parameters, we use model pruning technology, and the steps are 1. Channel pruning of the network. By setting an appropriate pruning rate, and according to the value of $\gamma$, the high-contribution channels are retained, and the low-contribution channels are deleted. 2. Layer pruning of the network. For each CBL and Block of the RPDnet4 backbone network, the average value of each layer is sorted, and then the layer with the smallest average value is selected for layer pruning. 3. After compressing the width and depth of the RPD network through steps 1 and 2, respectively, fine-tune the RPD network to restore the detection accuracy of the network model.

The data set used in this paper comes from the video images of Zhengzhou Xinzheng Airport, including different types of single-passenger aircraft, multiple occluded passenger aircraft, and other small target images, a total of more than 11,000 images, covering sunny, foggy, rainy, and other daytime weather conditions. The training, validation, and test sets are made according to 6:2:2.

### Ablation experiments

We perform a series of ablation experiments to understand better and analyze our key contributions’ impact. Table 2 shows that the PRD detection model adopts different image classification networks as the backbone. We validate our proposed model by comparing and analyzing model parameters, detection accuracy, and inference time, mainly since two modules (i.e., CBL and BLOCK) constitute the backbone network. We just changed the backbone network during the experiment, and other modules remained unchanged. The training strategy of the model remains the same, the data preprocessing steps are the same, and the initialization parameters are the same.

## Results and discussion

To effectively evaluate the performance of the network model, the precision P (Precision), the recall rate R (Recall), Inference time and the mean average precision mAP (mean Average Precision) are selected to evaluate the detection ability of the network model. The formula is as follows:

10 $$\begin{aligned} \mathrm {P}= \frac{\mathrm {TP}}{\mathrm {TP}+\mathrm {FP}} \end{aligned}$$

11 $$\begin{aligned} \mathrm {R}= \frac{\mathrm {TP}}{\mathrm {TP}+\mathrm {FN}} \end{aligned}$$

12 $$\begin{aligned} \mathrm {AP}= \int _{0}^{2} \mathrm {P}(\mathrm {R}) \end{aligned}$$

13 $$\begin{aligned} \mathrm {mAP}= \frac{\sum _{\mathrm {i}=1}^{\mathrm {C}} A P_{\mathrm {i}}}{\mathrm {C}} \end{aligned}$$

In the formula, TP represents the correct positive sample detection. For example, the aircraft in the image is detected as an aeroplane. FP represents the negative sample falsely detected as a positive sample. For example, the aeroplane is incorrectly identified as the background. FN represents the positive sample falsely detected as a negative sample, such as the background detected as an aeroplane. P(R) represents the curve drawn with the detection accuracy P , and the recall rate R, the area enclosed by this curve and the coordinate axis is the detection class accuracy AP; C represents the target category, mAP is an average of all categories.

This paper compares RPD with Faster-RCNN, SSD, YOLOX-Tiny, YOLOX-Nano and YOLOV3 from four aspects: mAP, FPS (Frame Per Second), Inference time and Param (Parameter), as shown in Table 1.

**Table 1:** Comparison of the algorithm performance.

Model	AP (%)	FPS (F/S)	Param (M)	Inference time (ms)
Faster-RCNN	82.3	1.2	350.8	25.7
SSD	67.7	2.5	117	8.6
YOLOV3	83.8	8.4	105	7.7
YOLOX-Tiny	79.4	38.6	17.4	1.3
YOLOX-Nano	73.6	55.2	8.7	0.6
RPD	88.6	13.6	69.6	5.1

Faster-RCNN uses VGGNet16 as the feature extraction network, uses the RPN (Region Proposal Network) network instead of the Selective Search method in R-CNN^51^ to generate regional proposal windows, and uses the non-maximum suppression algorithm to eliminate proposals with large overlapping areas. The window improves the quality of the proposed window, and the detection accuracy reaches 82.3%. However, Faster R-CNN is a two-stage target detection. The RPN network uses the sliding method to detect the process of generating multi-scale anchors, which is time-consuming, and the model parameters are the largest, reaching 350.8M.

SSD, YOLOV3, and RPD are all based on single-stage detection, directly classifying and regressing images, and the model parameters are significantly lower, 105M, 117M, and 69.6M in sequence. SSD eliminates the proposal generation phase and the subsequent feature resampling process. Its FPS is 2.5, the detection speed is two times faster than Faster R-CNN, and FPS is 2.5, but in terms of detection accuracy, mAP is 67.7% among all models, the detection accuracy is the lowest.

The SSD prediction target bounding box is an offset relative to the default bounding box position of the network. This prediction is not stable enough at the beginning of training. Yolov3 predicts the offset. The predicted result forces the output value between 0 and 1 through a sigmoid function, improving detection accuracy, reaching 83.8%. YOLOV3 is three times faster than SSD in terms of detection speed, and the FPS gets 8.4.

YOLOX-Tiny and YOLOX-Nano are two lightweight models of YOLOX. Although the model parameters are significantly reduced, only 17.4M and 8.7M, the detection accuracy is far from the detection requirements of airport air traffic.

The RPD model in this paper has the following characteristics:

(1)Feature extraction network module (RPDNet4): The core component CBL module uses 11 and 33 convolution kernels to increase the receptive field, separate the critical contextual features, and reduce the network running speed; Separate convolution [50] extracts feature maps. From experience, the effect is almost the same as standard convolution, and the computational cost is significantly lower than that of standard convolution. The RPDNet4 feature extraction network is better than VGG-16 and Darknet-53 in parameter quantity.

(2)Adjacent scale feature fusion and prediction module. Faster-RCNN only uses the feature map of the network’s last layer to predict the target. SSD tried to use the pyramid level feature of the convolutional neural network to predict the target, but it gave up Shallow features. Compared with SSD, YOLOV3 uses shallow features, splices different feature maps, increases the number of channels, and predicts targets at three scales, significantly improving the detection ability of small targets. The neck module in this paper adopts the adjacent scale feature fusion technology to fuse the features of different abstraction levels of layers (Fig. 2) and build a low-level to high-level feature pyramid structure (Neck1, Neck2, Neck3, Neck4 shown in Fig. 5), and then use the CBL module to eliminate the aliasing effect of upsampling to generate the feature map required by the Prediction module. The four scales of 160*160, 80*80, 40*40, and 20*20 in the Prediction module can share classification and regression parameters at all levels. This structure enables our mAP to reach 88.6%, higher than YOLOV3. 5.4%, which is 23.6% higher than SSD.

On the whole, compared with YOLOv3, Fast-RCNN, and SSD models, the detection accuracy of the RPD model is 88.6%, an increase of 5.4%, 7.1%, and 23.6%, respectively; in terms of model parameters, the RPD model is 69.6M, a decrease of 40.5%, 33.7%, and 80.2%, which can meet the real-time detection of airport aircraft.

Furthermore, we conduct an ablation study on the RPD model to assess our proposed technique’s performance, particularly the two modules (i.e., CBL and BLOCK) that constitute the backbone network. The ablation experiments for our suggested model RPD+RPDNet4 are practical, and the comparison results are provided in Table 2. The results show an improvement in accuracy for each example, showing that the RPD models are all useful. In the first layer of PRDNet4, the size of the convolution kernel of the first two CBL modules is 3*3, which expands the receptive field and textcolorredreduces the parameters as much as possible. The size of the convolution kernel of the CBL module of the remaining layers is 1*1. Upscaling the dimension of the channel greatly increases the nonlinearity while keeping the scale of the feature map unchanged (that is, without losing resolution). The Block module reduces the computational parameters of the feature extraction network as much as possible while ensuring the detection accuracy of the network model. Therefore, the CBL and Block modules can pay more attention to the intricate details of the image and obtain better detection results. In other words, it proves that the RPDNet4 feature extraction network preserves the most critical information in the image and suppresses the unnecessary information, resulting in more discriminative features for surface aircraft recognition. Combining these two modules yields the best results, demonstrating that our approach is feasible and beneficial.

**Table 2:** Comparison of results of ablation experiments.

Model	AP (%)	FPS (F/S)	Param (M)	Inference time (ms)
RPD + VGGNet16	77.3	2.6	123	15
RPD + VGGNet19	77.8	2.6	123.7	15
RPD + ResNet50	84.9	1.9	131	15.6
RPD + ResNet101	88.7	1.5	256	21.4
PD + ResNet152	90.3	1.1	380.8	27.9
RPD + DarkNet53	89.1	8.9	147	10.8
RPD + RPDNet4	88.6	13.6	69.6	5.1

**Figure 6:**
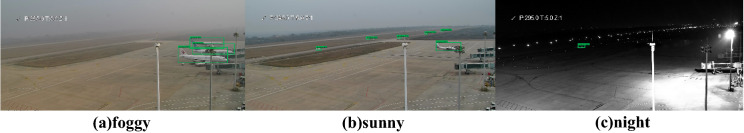
RPD detection renderings in different scenarios.

**Figure 7:**
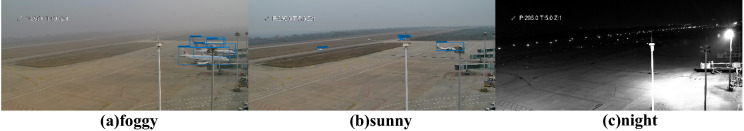
Faster-RCNN detection renderings in different scenarios.

**Figure 8:**
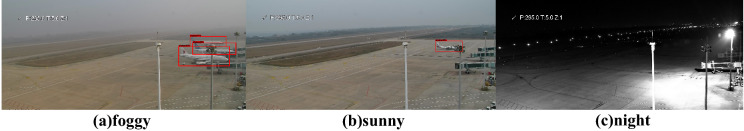
SSD detection renderings in different scenarios.

**Figure 9:**
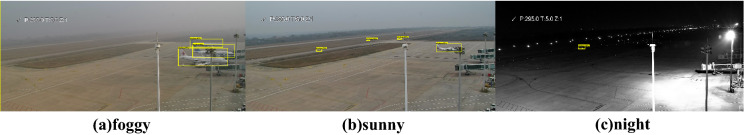
YOLOV3 detection renderings in different scenarios.

**Figure 10:**
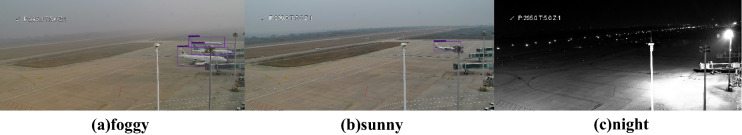
YOLOX-Tiny detection renderings in different scenarios.

**Figure 11:**
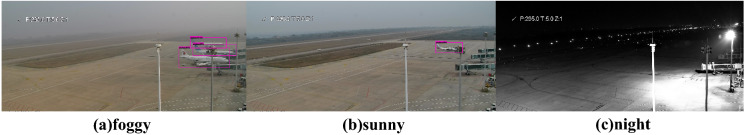
YOLOX-Nano detection renderings in different scenarios.

To further verify the effectiveness of the RPD model, the detection results of Faster-RCNN (Fig. 6), SSD (Fig. 7), YOLOV3 (Fig. 8), RPD (Fig. 9) and YOLOX-Tiny (Fig. 10) and YOLOX-Nano (Fig. 11) are visualized.

Comparing part (a) of the figures, we can find that SSD has missed detection because the model does not extract enough semantic information to distinguish the background. Faster-RCNN, YOLOV3, and RPD detection effects are sound; part (b) of the figure, YOLOV3 is better than Faster-RCNN, SSD, YOLOX-Tiny and YOLOX-Nano for small target detection, but some small targets have missed detection. RPD detects the most significant number of small targets and two more targets than YOLOV3 because our position loss function introduces GIou Loss. Simultaneously, the adjacent scale feature fusion technology is used to fuse the deep semantic information into the shallow features layer by layer, improving the detection accuracy of small objects. It can be found in part (c) that Faster-RCNN, SSD, YOLOX-Tiny and YOLOX-Nano can not recognize the aircraft in the airport night scene. Still, both YOLOV3 and RPD can recognize it, which verifies that the RPD detection model in this paper can effectively suppress the interference of complex environments.

## Conclusion

In this paper, we have solved the problem of poor identification of small target aircraft at airports in complex scenes, and it is challenging to meet the real-time detection task of airport air traffic control. We propose and build a lightweight object detection network model suitable for scene video planes. Firstly, the feature extraction backbone network RPDNet4 is designed using the depth separable convolution technology and residual module. The network model structure is composed of CBL module and Block module, which greatly reduces the network model parameters while ensuring the accuracy of target detection. Secondly, through the adjacent scale feature fusion module, a low-level to high-level feature pyramid structure is constructed, and the four-scale prediction module is used to share classification and regression parameters at all levels. Finally, the loss function of the RPD network model structure is optimized to reduce the actual deviation between the target frame and the real frame.

Compared with different target detection algorithms, the validity of the RPD network model structure in this paper is verified. In the future, the model proposed in this paper is planned to be used for aircraft tracking in busy airports with complex background changes, to try to solve the problem of poor recognition rate at night and to facilitate monitoring by air traffic controllers and flight crews.

## Data Availability

The data supporting this study’s findings are available from the corresponding author upon reasonable request.
